# Investigation of Arsenic-Stressed Yeast (*Saccharomyces cerevisiae*) as a Bioassay in Homeopathic Basic Research

**DOI:** 10.1100/tsw.2011.45

**Published:** 2011-03-07

**Authors:** Tim Jäger, Claudia Scherr, Ursula Wolf, Meinhard Simon, Peter Heusser, Stephan Baumgartner

**Affiliations:** ^1^ Institute of Complementary Medicine KIKOM, University of Bern, Switzerland; ^2^ Research Institute of Organic Agriculture, Frick, Switzerland; ^3^ Society for Cancer Research, Hiscia Institute, Arlesheim, Switzerland; ^4^ Institute for Chemistry and Biology of the Marine Environment, University of Oldenburg, Germany; ^5^ Center for Integrative Medicine, University of Witten/Herdecke, Germany

**Keywords:** Lemna gibba, duckweed, Saccharomyces cerevisiae, yeast, homeopathy, arsenic, Arsenicum album, nosode, gibberellic acid

## Abstract

This study investigated the response of arsenic-stressed yeast (*Saccharomyces cerevisiae*) towards homeopathically potentized Arsenicum album, a duckweed nosode, and gibberellic acid. The three test substances were applied in five potency levels (17x, 18x, 24x, 28x, 30x) and compared to controls (unsuccussed and succussed water) with respect to influencing specific growth parameters. Five independent experiments were evaluated for each test substance. Additionally, five water control experiments were analyzed to investigate the stability of the experimental setup (systematic negative control experiments). All experiments were randomized and blinded. Yeast grew in microplates over a period of 38 h in either potentized substances or water controls with 250 mg/l arsenic(V) added over the entire cultivation period. Yeast's growth kinetics (slope, Et_50_, and yield) were measured photometrically. The test system exhibited a low coefficient of variation (slope 1.2%, Et_50_ 0.3%, yield 2.7%). Succussed water did not induce any significant differences compared to unsuccussed water. Data from the control and treatment groups were both pooled to increase statistical power. In this study with yeast, no significant effects were found for any outcome parameter or any homeopathic treatment. Since in parallel experiments arsenic-stressed duckweed showed highly significant effects after application of potentized Arsenicum album and duckweed nosode preparations from the same batch as used in the present study, some specific properties of this experimental setup with yeast must be responsible for the lacking response.

## INTRODUCTION

The possibility of a specific homeopathic remedy effect is still discussed controversially. Despite the lack of a verified theoretical model, several randomized, placebo-controlled, double-blind clinical trials reported effects of homeopathic preparations superior to placebo[1,2,3,4]. Recent reviews of preclinical investigations also found some evidence for a specific drug action of homeopathic remedies[5,6,7,8], but reproducibility of the experiments still poses a problem[7,9,10].

Thus, the question of best-suited test systems and apt methodology in homeopathic basic research is open to this day. We aimed tot investigate the response of organisms of different organizational levels, namely multi- vs. unicellular, towards homeopathic preparations in simultaneous experiments under comparable conditions. Therefore, we searched for test systems that were fast and simple, thereby allowing large numbers of experimental replications and conditions, eliminating disadvantages such as placebo effect or ethical concerns, and that had been successfully used in previous homeopathic basic research studies. Based on the assumption that a characteristic feature of homeopathic preparations is to induce equilibrating and self-regulating effects, test systems with impaired organisms are to be expected to yield stronger effects after application of homeopathic preparations than test systems using healthy organisms. However, test systems with impaired organisms usually exhibit a considerable increase of variance[11,12]. Hence, major attention has to be given to a high degree of standardization in order to achieve standard deviations as low as possible.

Two models were chosen. The first model was a system with duckweed (*Lemna gibba* L.), a multicellular autotrophic water plant. The second model that met the requirements outlined was yeast, *Saccharomyces cerevisiae*, a unicellular heterotrophic organism. Both duckweed and yeast have often been used in standardized bioassays as research organisms[13,14,15,16]. Furthermore, unimpaired duckweed and yeasts have successfully been used in homeopathic basic research[17,18,19].

The stressor for inducing unbalanced conditions was chosen after an experimental comparison of copper sulfate ($CuSO_4$), arsenic(V), and UV-B radiation, which revealed arsenic(V) to exhibit the smallest variance in both test systems. After optimizing experimental conditions (e.g., degree of damage and point in time of measurement), several test substances were screened as homeopathic treatments to alleviate the stress induced. Effects of potentized Arsenicum album, a duckweed nosode, and gibberellic acid were investigated in additional experiments to assess reproducibility of the screening results. All experiments were performed with both bioassays (yeast and duckweed) in parallel (at the same day, at the same location, by the same experimenter, and with identical homeopathic preparations) in order to allow exact comparison of the results obtained. In addition, systematic negative control experiments were performed to control the stability of the experimental setup.

The results of the investigation with duckweed have already been published elsewhere[20,21]. In this publication, we present the data obtained with the yeast bioassay and compare the results of both bioassays. Reporting of the study was adapted to the latest guidelines for experimental basic research in homeopathy[22].

## MATERIALS AND METHODS

### General Experimental Design

The experimental investigations were performed in two parts. In a primary screening, a total of 12 experiments were performed, 11 with different potentized substances (Table 1) and one systematic negative control experiment. Because we did not observe any significant effects on the growth curve parameters for *S. cerevisiae* in the 11 screening experiments, for the additional independent replication experiment, we selected only substances that had yielded significant effects in the screening experiments of the *Lemna* test system (Arsenicum album, a duckweed nosode, and gibberellic acid). We subsequently performed four additional independent experiments for each of the three substances and each test organism,

**TABLE 1**
**Substances Included in the Screening Experiments**

| Substance, Empirical Formula | Category | Source | Concentration of Potency Stock Preparation |
|---|---|---|---|
| Arsenicum album ($As_2O_3$) | Metal | Weleda, Arlesheim, Switzerland | 5x, trituration |
| Gibberellic acid; $C_{19}H_{22}O_6$ | Plant hormone | Sigma-Aldrich, Buchs, Switzerland | 10 mg/1000 ml |
| Nosode | Plant digestion | Own laboratory | 100 g/900 ml |
| Arsenic(V) (sodium dibasic arsenate 7-hydrate; $AsHNa_2O_4$ x $7H_2O$) | Metal | Sigma-Aldrich, Buchs, Switzerland | 100 g/900 ml |
| Phosphorus | Nutrient | Weleda, Arlesheim, Switzerland | 3x, dilution |
| Conchae | Oyster shell | Weleda, Arlesheim, Switzerland | 100%, powder |
| Acidum picrinicum | Explosive agent | Remedia, Eisenstadt, Austria | 3x, dilution |
| Argentum nitricum | Metal | Weleda, Arlesheim, Switzerland | 10x, trituration |
| Crotalus horridus | Venom | DHU, Karlsruhe, Germany | 6x, dilution |
| Hepar sulfuris | Oyster shell and flower of sulfur | Weleda, Arlesheim, Switzerland | 1x, trituration |
| Mercurius vivus naturalis | Metal | Weleda, Arlesheim, Switzerland | 3x, trituration |

designed as identical repetitions of the initial screening experiment (Fig. 1). Furthermore, we conducted four additional full-size experiments with pure water as the only treatment parameter (systematic negative control experiments) to investigate the stability of the experimental setup over the entire study period. For the final statistical evaluation, the data from the screening experiment were pooled with those of the four repetition experiments. Thus, a total of 20 experiments (four experimental series with five independent experiments each) entered the final data evaluation. All experiments were carried out between January and September 2009.

## Preparation of Potentized Test Solutions and Controls

A detailed description of the sample preparation has been given in a precursor publication[21]. All test solutions for one experiment (potencies and controls) were freshly prepared according to the multiple glass method between 6 a.m. and 9 a.m. on the day of the experiment from the same batch of distilled and autoclaved water.

For preparation of the nosode, duckweed was grown in 2000 ml of moStM (see below) comprising 158 mg/l arsenic(V) for 48 h. Duckweed was cut into small pieces, given into 85 ml of distilled water and 15 ml of ethanol (94%, Alcosuisse-S15-sekunda, Schachen, Switzerland) and agitated for 2 h (Turbula T2 C, Willy A. Bachofen AG, Basel, Switzerland) in an Erlenmeyer flask of Duran® glass (250 ml, Schott, Mainz, Germany). After maceration at 20°C in diffused light for 21 days, the extract was filtered (Macherey-Nagel, MN-619-eh ¼ Ø 185 mm, Germany) and stored at 4°C (Thermostat cabinet TS 606/3, WTW GmbH, Weilheim, Germany) for 12 days. Gibberellic acid (Sigma-Aldrich, Buchs, Switzerland) was potentized in acetone (AppliChem A2300 Darmstadt, Germany) for reason of solubility to 1x and further on in distilled water. Arsenicum album was obtained in the lowest potency available (5x, Weleda, Arlesheim, Switzerland). All samples were further potentized in distilled water (Büchi, Fontavapor-250, Flawil, Switzerland). Detailed information regarding the preparation of the other potentized substances used in the screening experiments (Table 1) can be found elsewhere[21].

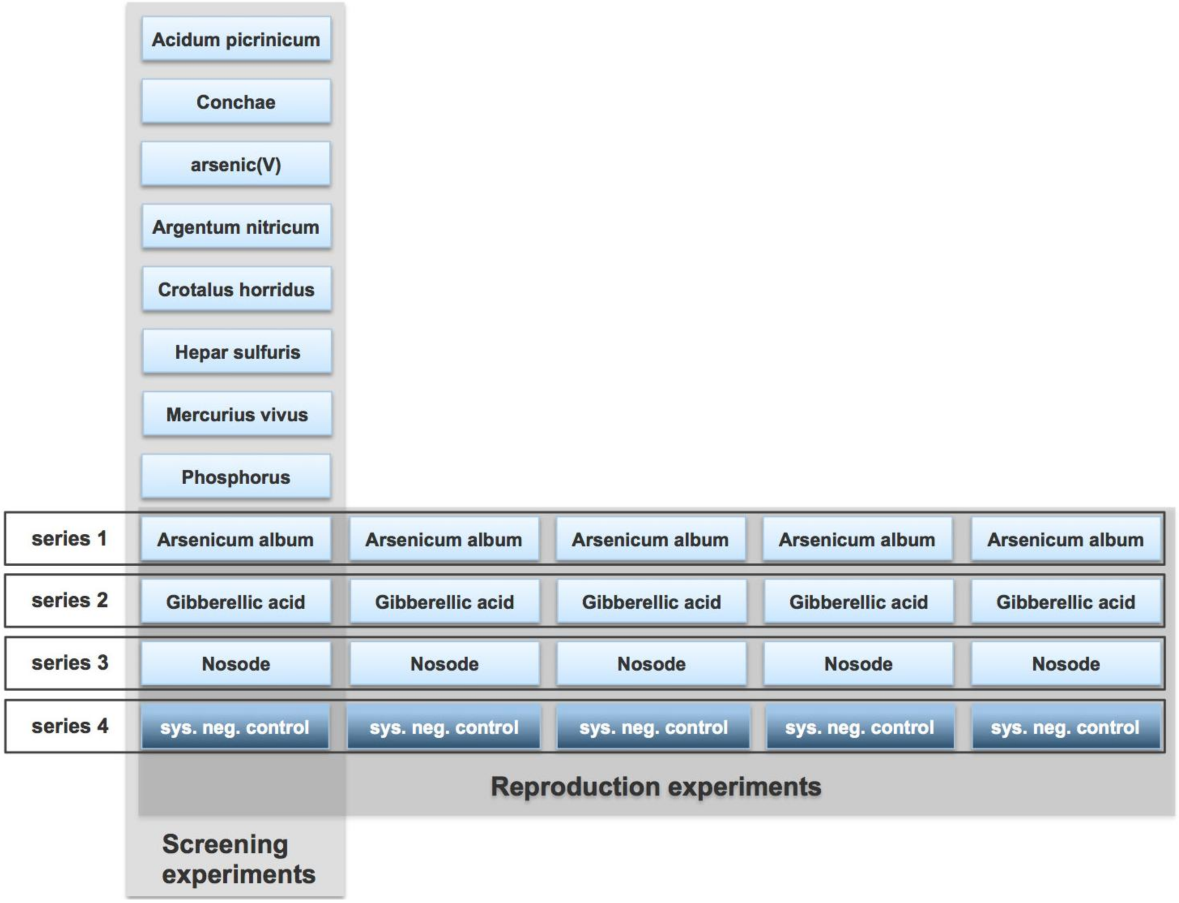

**FIGURE 1.** Overview of all analyzed experiments. One box corresponds to one single experiment. The data evaluation of the four main experimental series (1–4) comprised five independent experiments each. (sys. neg. control = systematic negative control experiment).

For the potentization process, Erlenmeyer flasks of Duran® glass (≤6x: 250 ml, ≥7x: 500 ml, Schott, Mainz, Germany) were used. Then, 15 ml of potency stock solution was added to 135 ml of distilled water. The flask was agitated once upside down to generate a vortex. After calming the vortex, the flask was shaken a second time, producing a chaotic agitation in the water. These two steps were repeated ten times. For the next potency level, 15 ml of this solution were added to the next potentization vessel containing 135 ml of distilled water and agitated in the same manner. At potency level 7x, flask size was changed from 250 to 500 ml, and the filling volume rose to 350 ml; thus, 35 ml of the former potency level were added to 315 ml of distilled water. This process of successive tenfold dilution steps and vigorous shaking proceeded until potency step 33x was accomplished.

Two types of controls were prepared: unsuccussed water (c0) and succussed water (c1), corresponding to water 1x, shaken analogously to the potencies described above. Two samples of unsuccussed water were prepared in 500-ml Erlenmeyer flasks and three samples of succussed water in analogous Erlenmeyer flasks. These controls were chosen because comparison of unsuccussed and succussed controls allows estimation of the influence of the unspecific physicochemical effects induced by agitation[23].

From the potencies prepared, five potency levels (17x, 18x, 24x, 28x, 30x) were used in the yeast bioassay. Together with five control preparations (two samples unsuccussed and three samples succussed water), 10 samples were prepared in total for yeast. All test solutions were randomized and coded

(blinded) by a person not involved in the experiments by manual random assignment of a double-letter code from a predefined list.

## General Experimental Procedure

A single yeast experiment comprised 96 wells of a microplate inoculated with *S. cerevisiae* (Fig. 2). There were n = 12 experimental parameters in total, n = 10 letter-coded samples (potencies and controls, see below) with impaired yeast and two open control conditions with unimpaired yeast. The latter two controls were only used for calculation of the degree of damage induced by arsenic(V). For every parameter, eight replicates were used (12 parameters × 8 replicates = 96 wells) and randomly allocated in a fixed-blocked randomization scheme (Fig. 2B). The 10 coded samples consisted either of five potency levels (17x, 18x, 24x, 28x, 30x) of a given substance and of five independent control preparations (two samples unsuccussed water and three samples one-time succussed water), or – in the case of the systematic negative control experiments – of 10 unsuccussed water samples coming from the same source. Yeast was stressed with arsenic(V) over the entire experimental period for 38 h and grew in either potentized substances or water controls. Growth kinetics were determined by measuring optical density at 600 nm every hour for a period of 30 h, yielding a sigmoid growth curve; multiple regression was used to calculate slope, $Et_{50}$, and yield (see below).

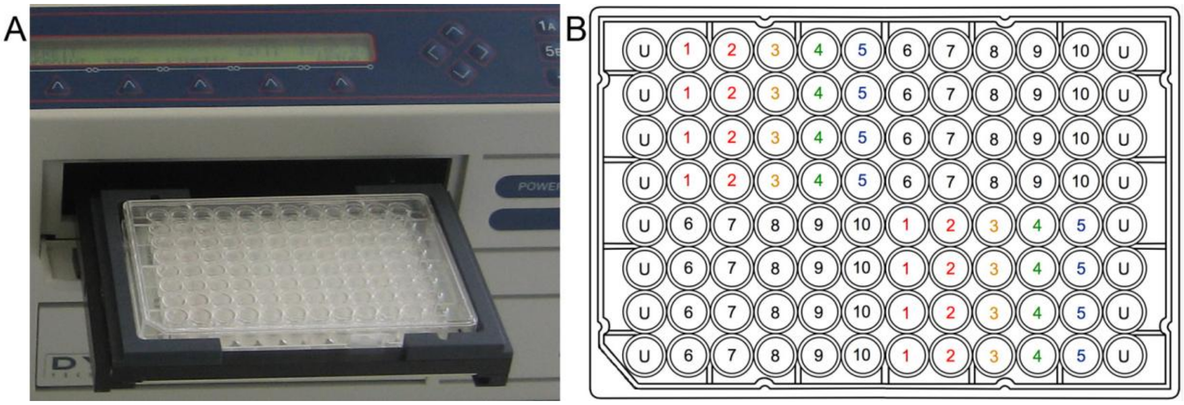

**FIGURE 2**. (A) Microplate with 96 wells in the drawer of the automated microplate reader. (B) Spatial arrangement of the ten coded samples on the microplate, as measured in one experiment (five potency levels of a given substance and five water controls [three succussed and two unsuccussed]). In every verum experiment, the coded samples were newly randomly allocated to the fixed blocks. In the case of systematic negative control experiments, samples 1–10 were of identical origin (distilled water). Wells of both outer columns (U) were filled with unimpaired yeast (without arsenic[V]). These uncoded samples were used for calculation of the degree of damage only and did not enter the statistical analysis of the coded samples.

## Yeast Cultivation and Measurement

The yeast species *S. cerevisiae* X2180 (MATa/α SUC2/SUC2 mal/mal mel/mel gal2/gal2 CUP1/CUP1) was used. For long-time storage, several separate cultures were harvested and resuspended in 15% (w/v) glycerol (Appli Chem, Darmstadt, Germany). Then the cultures were dispensed into 1.2-ml aliquots (Cryovial T310-1A, Simport Plastics, Quebec, Canada) and frozen at –80°C (Revco Ultima II ULT 1790-9-V31, Asheville, USA). Yeast from these storage cultures was taken every 3 weeks, each time from a new aliquot, then it was grown on Universal medium for yeast 186 (YM 186) (German Collection of Microorganisms and Cell Cultures, Braunschweig, Germany), which contained: 3 g/l yeast extract

(Oxoid, Basingstoke, Great Britain), 5 g/l proteose peptone (Oxoid), 3 g/l malt extract (Oxoid), 10 g/l Bacto® dextrose (Difco, Detroit, USA), and 15 g/l agar bacteriological No. 1 (Oxoid). The pH was adjusted with HCl (Fluka, Buchs, Switzerland) to 5.9 ± 0.1. After growing for 2 days in Petri dishes at 30°C (Thermostat cabinet TS 606/3), yeast was stored at 4°C (Thermostat cabinet TS 606/3) for a period of 14 days. Subsequently, it was transferred to new YM 186 medium.

Before an experiment was carried out, one yeast colony was added to 20 ml of liquid Yeast N base medium (containing 6.7 g/l DifcoTM yeast nitrogen base [Becton, Dickinson, Sparks, USA] and 5 g/l Bacto® dextrose [Difco], pH 5.4 ± 0.1) in culture tubes (culture tube, 18 × 180 mm, Schott Duran, Mainz, Germany) and was grown at 30°C for 16 h under permanent agitation. Turbidity was measured (Turbidity Meter, ESD Engineered Systems and Designs, Newark, USA) in order to control that the log phase was reached. Then, the yeast cells were used as inoculum for the growth kinetic measurements, which were conducted on Yeast N base medium agar (Yeast N base medium and 6 g/l agar bacteriological No. 1 [Oxoid]).

Sterile Yeast N base medium agar (200 µl) was filled in the gaps between the wells of a sterile microplate to avoid potential cross-over contamination between treated samples and water controls. Then, 10 ml of the samples were sterile filtered (Sterile Syringe Filter w/0.2 µm Cellulose Acetate Membrane, VWR, USA; Syringe, BD DiscarditTM II 20ml, Fraga, Spain) under laminar flow (Skan AG, Basel, Switzerland) and filled in reservoirs (Reagent reservoirs V-shape hinged lid Polypropylene 175ml, Socorex, Lausanne). Sample (100 µl) was added to the wells of a sterile 96-well microplate (Multiple Well Plate 96, Well flat bottom with lid, Sarstedt, Newton, USA) in a blocked randomization scheme for all experiments. The columns at the edge (2 × 8 wells) were filled with Yeast N base medium agar without arsenic. The remaining 80 wells were filled with 10 coded samples, either potentized substances or controls, in eight replicates each, in two blocks of four adjacent wells. Samples were pipetted with a multiple-channel pipette (Research pro 8-channel 20–300 µl, Eppendorf, Hamburg, Germany) as two times 4 × 100 µl. Then, 100 ml of sterile Yeast N base medium agar, including 250 mg/l arsenic(V) ($AsHNa_2O_4$ x $7H_2O$, Sigma-Aldrich, Buchs, Switzerland), was tempered at 39.0°C before yeast was added (about 60,000 cells/ml yeast cells). The mixture was agitated for 3 sec and filled in a reservoir (Reagent reservoirs V-shape hinged lid Polypropylene 175 ml). Next, 150 µl were immediately pipetted in each well (Research pro 8-channel 20–300 µl), resulting in a final concentration of about 36,000 cells/ml in each well. For growth kinetic measurements, the microplate was put in a microplate reader (MRX II TC, Dynex Technologies, Chantilly, USA) at 30°C, which in turn was installed in an incubation chamber (Thermostat cabinet TS 606) at 24°C in order to improve temperature stability. The optical density ($I_0/I$; intensity of incident light/intensity of transmitted light) was measured at a wavelength of 600 nm every hour from the $8^{th}$ hour until the $38^{th}$ hour. For data reduction, a regression with four parameters was fitted to the yeast growth data individually for each well of the microplate, according to the equation: optical density (OD) = bottom + ((top – bottom) / (1 + 10 exp (($Et_{50}$-time) × slope))) (Fig. 3). Three outcome parameters were finally used for statistical analysis: slope [$h^{-1}$], yield (top – bottom), and $Et_{50}$ [h]. Any materials used in further experiments were cleaned with tap water, thoroughly rinsed three times in running demineralized water (>0.5 µS/cm, Christ Milistil P-24, Christ Aqua Ecolife, Aesch, Switzerland), once in cold distilled water, and then dried for 70 min at 95°C. On the day of experiments, they were autoclaved at 121°C for 15 min (GE 406, GETINGE AB, Getinge, Sweden).

## Statistical Analysis

Regarding a possible succussion effect, data of the unsuccussed (c0) and succussed water controls (c1) of experiments with potentized substances were analyzed using a two-way analysis of variance (ANOVA) *F*-test for independent samples. Data from the systematic negative control experiments were not used since they included only unsuccussed water.

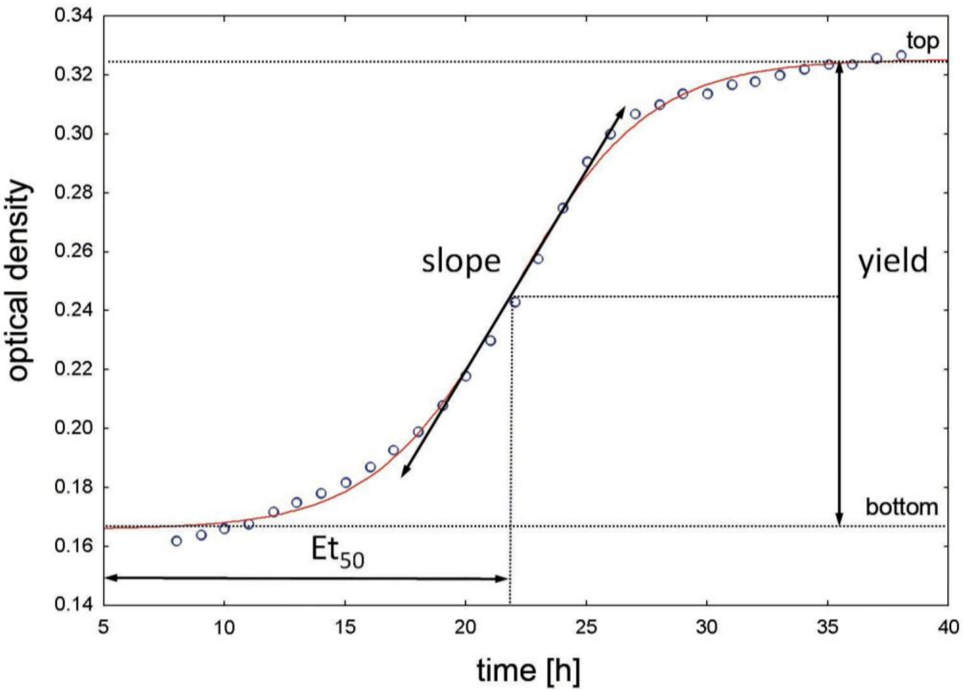

**FIGURE 3.** Parametrization of the *S. cerevisiae* growth curve. A four-parameter regression (top, bottom, slope, $Et_{50}$) was fitted to the measured yeast growth data (empty circles) for each well of the microplate.

In the screening experiments, differences in growth data between pooled potencies and pooled water controls (succussed and unsuccussed) were evaluated for statistical significance based on *t*-tests for independent samples. In all statistical analyses, the level of significance was $\alpha = 0.05$.

Data from the five systematic negative control experiments were used to estimate the intraexperimental variability of the bioassay. We grouped the data from the 80 wells of every single experiment into 10 groups of eight replicates and calculated mean values for these 10 subgroups for the outcome parameters slope, $Et_{50}$, and yield. Based on these 10 values, the coefficient of variation was calculated for every single experiment and outcome parameter.

A comparison of growth data ($Et_{50}$ and yield) between pooled potencies and pooled water controls (succussed and unsuccussed) was evaluated for statistical significance based on two-way ANOVA *F*-tests for independent samples. An interaction term between experiment number and treatment was included in the statistical model in order to get some information regarding reproducibility and possible effect modulating factors associated with the date of the experiment. In order to compare the five experiments of one test substance, data of each experiment were normalized to the pooled water controls.

The statistical analysis of the systematic negative control experiments with yeast was performed absolutely identical to the experiments with potentized substances (see below) by using the randomization lists from the verum experiments (with Arsenicum album, nosode, and gibberellic acid).

Furthermore, the hypothesis of a decrease of variability after application of homeopathic potencies[24,25,26,27] was tested. Levene's test was conducted to test homogeneity of variances. Data distribution was evaluated graphically by quantile-quantile plots. All data were analyzed using the software STATISTICA Version 6 (Stat Soft, Inc. Tulsa, USA).

From all experimental series, a total of 4800 (20 experiments × 80 wells × 3 observation parameters) final outcome data points was obtained. Since some singular outliers were observed (up to 100 × standard deviation), we decided to eliminate extreme values (cut-off >3.0 × SD, corresponding to 27 data points = 0.6% of all data). Thus, 4773 data points (99.4%) were integrated into the final statistical analysis.

## RESULTS

On average, arsenic-treated yeast exhibited a growth of approximately 65% for slope, 64% for $Et_{50}$, and 68% for yield compared to yeast growing without arsenic.

None of the 11 different potentized substances (Table 1) tested in the screening showed a significant effect on the growth kinetics ($Et_{50}$, slope, and yield) of *S. cerevisiae* (Table 2) when compared to the pooled water controls.

**TABLE 2**
**Statistical Analysis of the Screening Experiments with Impaired *S. cerevisiae*: Comparison (*t*-test) of Pooled Controls (c0, c1) and Pooled Potencies (17x, 18x, 24x, 28x, 30x) for Three Growth Curve Parameters for All Potentized Substances Tested**

| Experimental Run | p Values for Growth Curve Parameters | | | | | |
|---|---|---|---|---|---|---|
| | Slope | | $Et_{50}$ | | Yield | |
| Arsenic(V) | 0.415 | + | 0.79 | + | 0.108 | - |
| Hepar sulfuris | 0.315 | - | 0.97 | - | 0.567 | - |
| Mercurius | 0.869 | + | 0.67 | + | 0.477 | + |
| Phosphorus | 0.215 | - | 0.415 | - | 0.614 | + |
| Conchae | 0.248 | - | 0.529 | - | 0.955 | - |
| Acidum picrinum | 0.895 | - | 0.973 | - | 0.261 | + |
| Argentum nitricum | 0.856 | - | 0.169 | - | 0.998 | - |
| Crotalus horridus | 0.241 | - | 0.323 | - | 0.077 | + |
| Arsenicum album | 0.279 | + | 0.417 | + | 0.803 | - |
| Nosode | 0.481 | - | 0.875 | + | 0.992 | - |
| Gibberellic acid | 0.387 | + | 0.964 | + | 0.678 | - |

Includes *p* values of *t*-tests for independent samples; + : potencies numerically increased growth, – : potencies numerically decreased growth.

In ANOVA *F*-tests of yeast growth data of the three main experimental series, no significant main succussion effect and no significant interaction of succussion with experiment number were observed for any outcome parameter (Table 3). Therefore, data from both control groups were pooled for further analysis (defined as control c).

Yeast growth data (slope, $Et_{50}$, and yield) for the three main experimental series (Arsenicum album, nosode, and gibberellic acid) were analyzed separately, always in full two-way ANOVA with the independent variables treatment (n = 2, pooled data from all potency levels vs. pooled data from both control groups) and experiment number (1–5). The analysis yielded no significant main treatment effects for the three potentized substances regarding all three growth parameters (slope, $Et_{50}$, and yield; Table 4, Fig. 4A–C). For the duckweed nosode preparation, there was some trend for a treatment effect in the outcome parameter yield. Significant interactions of experiment number and treatment were observed for the growth parameter $Et_{50}$ in the Arsenicum album experiments and for the growth parameter yield in the experiments with gibberellic acid (Table 4). Levene's test was not indicative for any inhomogeneity of variances and quantile-quantile plots showed a normal data distribution (data not shown).

**TABLE 3**
**Comparison of the Effects of Unsuccussed (c0) and Successed Water Controls (c1) on the Three Growth Curve Parameters (Slope, $Et_{50}$, and Yield) of *S. cerevisiae***

| Series | Effects | p Values (F-test) for Growth Curve Parameters | | |
|---|---|---|---|---|
| | | Slope | $Et_{50}$ | Yield |
| Arsenicum album | 1: Experiment number | 1.000 | 1.000 | 1.000 |
| | 2: Treatment | 0.266 | 0.159 | 0.186 |
| | 1/2: Interaction | 0.679 | 0.379 | 0.284 |
| Nosode | 1: Experiment number | 1.000 | 1.000 | 1.000 |
| | 2: Treatment | 0.686 | 0.416 | 0.593 |
| | 1/2: Interaction | 0.366 | 0.905 | 0.906 |
| Gibberellic acid | 1: Experiment number | 1.000 | 1.000 | 1.000 |
| | 2: Treatment | 0.194 | 0.577 | 0.175 |
| | 1/2: Interaction | 0.109 | 0.097 | 0.263 |

ANOVA main effect and interaction *F*-tests were calculated for the three main experimental series (test substances Arsenicum album, nosode, and gibberellic acid) with five independent experiments for each test substance. Data were normalized to the mean of the pooled water controls for each individual experiment.

The global ANOVA *F*-tests yielded no significant main treatment effects for any outcome parameter calculated in the water control experiments (Table 4, series SNC, systematic negative control experiments). There was a trend for significance for the outcome parameter slope for the gibberellic acid randomization. In addition, there were two significant interaction effects between experiment number and treatment. The systematic negative control experiments revealed small coefficients of variation for all measured outcome parameters of the yeast test system (slope 1.17%, $Et_{50}$ 0.32%, yield 2.68%).

## DISCUSSION

To the best of our knowledge, this study is the first to investigate homeopathic preparations with two different bioassays in parallel experiments, i.e., at the same day, at the same location, by the same experimenter, and with homeopathic preparations from the same batch, freshly prepared on the day of the experiments.

We investigated in five independent experiments each whether the growth of yeast (*S. cerevisiae)* and duckweed (*L. gibba* L.) could be influenced by homeopathic preparations of Arsenicum album, a duckweed nosode, or gibberellic acid, compared to unsuccussed or succussed (1x) water. In the experiments with *S. cerevisiae*, we used the potency levels 17x, 18x, 24x, 28x, 30x; in those with *L. gibba*, we additionally included the potency levels 21x–23x and 33x. The results of the investigation with duckweed have already been published elsewhere[21,28].

In the screening experiments, no significant effects were observed for any homeopathic preparation tested in the yeast bioassay. In contrast to yeast, significant effects were observed in the screening experiments with *L. gibba*: homeopathic preparations of Arsenicum album, duckweed nosode, gibberellic acid, and Argentum nitricum induced significant effects on the growth rate of duckweed[21].

As in the experiments with duckweed, no significant succussion effect and no significant interaction of succussion with experiment number were observed for any outcome parameter of yeast growth data. Since succussed water (c1) did not differ from unsuccussed water (c0) in its effects on growth, we concluded

**TABLE 4**
**ANOVA *F*-Test Analysis of the Four Main Experimental Series***

| Series | Statistical Parameters | p Values for Growth Kinetics | | |
|---|---|---|---|---|
| | | Slope | $Et_{50}$ | Yield |
| Arsenicum album | 1: Experiment number | 0.606 | **0.034** | 0.893 |
| | 2: Treatment | 0.157 | 0.403 | 0.894 |
| | 1/2: Interaction | 0.606 | **0.034** | 0.893 |
| Nosode | 1: Experiment number | 0.453 | 0.391 | 0.280 |
| | 2: Treatment | 0.281 | 0.105 | 0.066 |
| | 1/2: Interaction | 0.453 | 0.391 | 0.280 |
| Gibberellic acid | 1: Experiment number | 0.991 | 0.357 | **0.017** |
| | 2: Treatment | 0.639 | 0.623 | 0.098 |
| | 1/2: Interaction | 0.991 | 0.357 | **0.017** |
| SNC (Arsenicum album) | 1: Experiment number | 0.485 | 0.268 | 0.307 |
| | 2: Treatment | 0.757 | 0.698 | 0.239 |
| | 1/2: Interaction | 0.485 | 0.268 | 0.307 |
| SNC (Nosode) | 1: Experiment number | 0.813 | 0.345 | **0.040** |
| | 2: Treatment | 0.614 | 0.335 | 0.962 |
| | 1/2: Interaction | 0.813 | 0.345 | **0.040** |
| SNC (Gibberellic acid) | 1: Experiment number | **0.031** | 0.449 | 0.778 |
| | 2: Treatment | 0.050 | 0.992 | 0.536 |
| | 1/2: Interaction | **0.031** | 0.449 | 0.778 |

* Test substances Arsenicum album, nosode, and gibberellic acid, as well as the systematic negative control experiments (SNC) evaluated with the three randomization lists from the verum experiments, with the independent parameters experiment number (n = 5, independent experiments) and treatment (n = 2, potencies vs. controls). Dependent variables (outcome parameters) were the three parameters slope, $Et_{50}$, and yield of the growth curve of *S. cerevisiae*. Data for the five potency levels (17x, 18x, 24x, 28x, 30x) and the five control samples (two samples unsuccussed water, three samples succussed water) were pooled. Data were normalized to the mean of the pooled water controls for every individual experiment. Significant values ($p < 0.05$) are shown in bold.

that possible unspecific effects due to the succussion procedure (e.g., increased ion dissolution from the glass vessel walls, pH changes due to changes in $CO_2$ concentration, etc.) were negligible in both bioassays. Therefore, effects of potentized substances (see below) were compared to the pooled data from both control groups (defined as control c).

Coefficients of variation were in the same order of magnitude for both bioassays. For duckweed growth rate, values were 0.7–1.0% ($r_{area}$ days 0–2: 1.03%, days 2–6: 0.76%, days 0-6: 0.78%; $r_{number}$ days 0–2: 0.89%, days 2–6: 0.98%, days 0-6: 0.68%). In the yeast bioassay, values were lower for $Et_{50}$ (0.32%) and higher for slope (1.17%) as well as yield (2.68%).

The statistical analysis of the systematic negative control experiments was performed absolutely identical to the experiments with potentized substances by using the randomization lists from the verum experiments (with Arsenicum album, nosode, and gibberellic acid). For the yeast bioassay, the global ANOVA *F*-tests yielded no significant main treatment effects for the outcome parameters analyzed. There was, however, a trend for significance for the outcome parameter slope for the gibberellic acid randomization, and two significant interaction effects between experiment number and treatment. These results

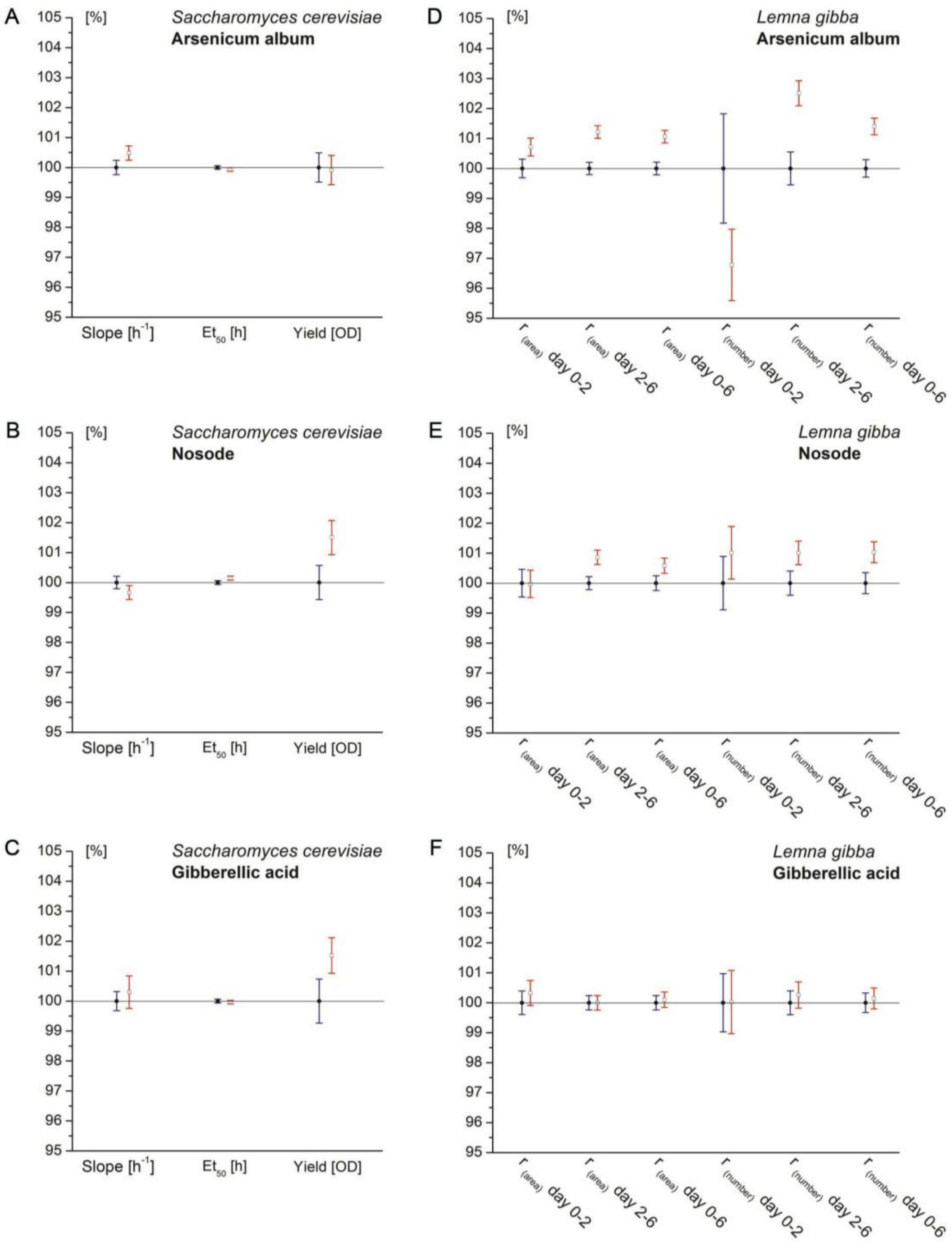

**FIGURE 4.** Growth kinetics of *S. cerevisiae* (slope, $Et_{50}$, and yield; A–C) (%) and *L. gibba* L. ($r_{(area)}$ and $r_{(number)}$ for days 0–2, 2–6, and 0–6; D–F) (%) (mean ± standard error; empty squares: potencies, black dots: controls) treated with different homeopathic preparations: (A,D) Arsenicum album; (B,E) duckweed nosode; (C,F) gibberellic acid. For yeast, data of the five potency levels investigated (17x, 18x, 24x, 28x, 30x) were pooled and compared to the pooled data of the five control samples set to 100% (two samples unsuccussed water, three samples succussed water). For duckweed, data of the nine potency levels investigated (17x, 18x, 21x–24x, 28x, 30x, 33x) were pooled and compared to the pooled data of the nine control samples set to 100% (four samples unsuccussed water, five samples succussed water). All three experimental series (A/D, B/E, C/F) comprised five independently performed experiments, respectively.

point to some weak systematic errors (e.g., due to spatial gradients in temperature). Due to the independent randomization applied in all experiments, these systematic errors were negligible regarding the main treatment effect. Consequently, the significant interactions of experiment number and treatment observed in the verum experiments ($Et_{50}$ for Arsenicum album and yield for gibberellic acid) have to be interpreted with great caution. The analysis of the systematic negative control experiments suggests that these effects should not be interpreted as treatment effects varying with time, but as randomized weak systematic errors. For duckweed, the systematic negative control experiments yielded no evidence whatsoever for systematic errors.

As in the screening experiments, we did not observe any effects of the homeopathic preparations in the repetition experiments using the yeast bioassay. In contrast, the growth rate of impaired *L. gibba* was enhanced after application of homeopathic potencies of Arsenicum album and nosode also in the repetition experiments[28]. Application of potentized Arsenicum album yielded the largest effects compared to water controls for the outcome parameters frond area (growth rate $r_{(area)}$ days 2–6: $p < 0.001$, days 0–6: $p < 0.001$) and frond number (growth rate $r_{(number)}$ days 2–6: $p < 0.001$, days 0–6: $p < 0.001$, Fig. 4D). Application of potentized nosode preparations also yielded significant effects on duckweed's frond area and frond number (growth rate $r_{(area)}$ for days 2–6: $p < 0.01$; growth rate $r_{(number)}$ for days 0–6: $p = 0.036$, Fig. 4E). Since the interaction between treatment and experiment number was not significant, the effects of potentized Arsenicum album and nosode seemed to be reproducible (within the limits of statistical power). Potencies of gibberellic acid did not exert any significant effects (Fig. 4F). Growth rates in the first time interval (days 0–2) were not influenced by any homeopathic treatment. Since the systematic negative control experiments did not yield any evidence for systematic errors associated with the experimental setup, the effects of homeopathic potencies of Arsenicum album and nosode on duckweed growth of impaired duckweed can be considered as reliable.

Regarding the activity of single potency levels, there were no obvious similarities in the results of the yeast and duckweed bioassays. As an example, the outcome parameters yield (showing some treatment trends for yeast, see Fig. 4) and growth rate $r_{(area)}$ days 2–6 for duckweed were compared in Fig. 5.

While duckweed clearly showed an increased growth rate after application of certain homeopathic preparations, yeast was not influenced by any of the preparations chosen. Possible reasons for this difference in behavior are discussed in the following.

Both experimental setups (arsenic[V]-impaired duckweed and arsenic[V]-impaired yeast) exhibited comparable small coefficients of variation (<3%) for all growth parameters. Correspondingly, it was possible to detect even quite small effects of the tested homeopathic preparations in the duckweed bioassay, in the screening as well as in the replication experiments. Since the coefficient of variation was comparable for the yeast and duckweed test systems, the lack of response of the yeast system cannot be due to lacking statistical power.

Neither duckweed nor yeast reacted to succussed water, compared to unsuccussed water. Thus, unspecific effects induced by the succussion step, such as increased ion dissolution from the glass vessel walls, pH changes due to changes in $CO_2$ concentration, air dissolution or suspension, radical formation, etc.[23,29], were irrelevant in both bioassays and can be excluded as reasons for the difference in the observed effects.

The systematic negative control experiments yielded some indication that minor systematic errors might have occurred in the yeast bioassay, while the duckweed bioassay was free of any systematic disturbing influences. For the yeast bioassay, the systematic errors were negligible regarding the main ANOVA treatment effects due to the independent randomization applied in all experiments. Since the coefficients of variation of the yeast outcome parameters were in the same order of magnitude as those of the duckweed assay, the differences in assay response were not caused by the minor systematic errors of the yeast assay.

Among the potentized substances investigated, two were chosen specifically for the *Lemna* test system: gibberellic acid and the nosode preparation (produced with *L. gibba*). Since the nosode was produced with arsenic-treated duckweed, it may seem implausible that this preparation might induce some reactions in yeast cells. However, biochemical reactions after arsenic stress might be similar in both organisms as they are both eukaryotic. Other substances of the screening were chosen specifically for yeasts, e.g., phosphorus due to a significant effect on healthy yeast (*Schizosaccharomyces pombe*)[19].

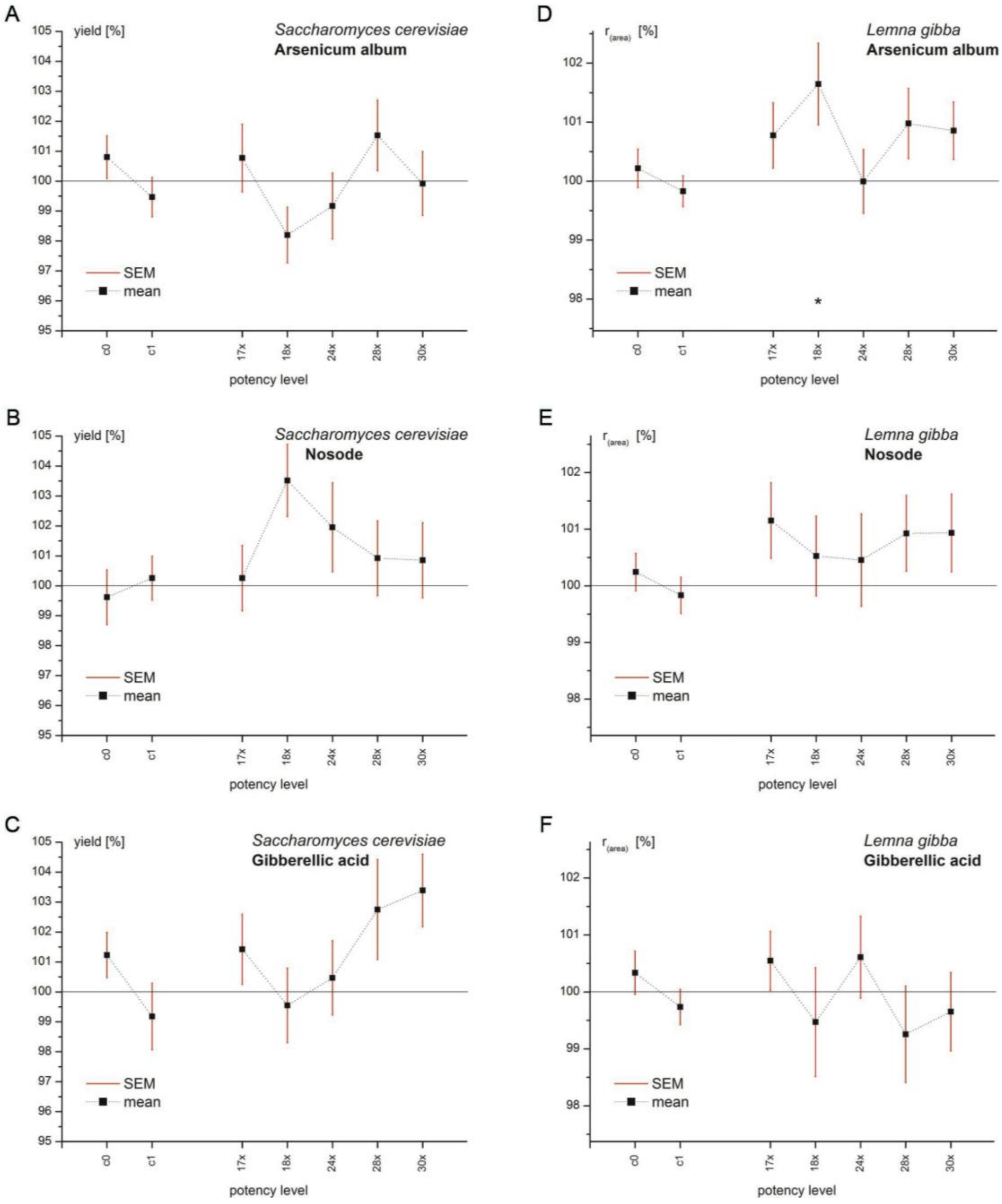

**FIGURE 5.** Growth kinetics of *Saccharomyces cerevisiae* (yield) (%) and area-related specific growth rates ($r_{(area)}$ days 2–6) (%) of *L. gibba* growing in different potency levels of selected test substances (A–C, D–F) in comparison to the corresponding water controls (c0 + c1). Mean values (dots) ± standard error for five independent experiments, respectively. Every data point for a single potency level is a mean from five independent experiments, with either eight replicates (wells) each for yeast (n = 40 per data point) or five replicates (beakers) each for duckweed (n = 25 per data point plotted). For yeast, the two data points for controls are an average from five independent experiments with 24 wells (succussed controls) or 16 wells (unsuccussed controls) (n = 120 and n = 80 per data point plotted). For duckweed, the two data points for the controls are an average from five independent experiments with 25 beakers (succussed controls) or 20 beakers (unsuccussed controls) (n = 125 and n = 100 per data point plotted). Data were normalized to the experimental mean of succussed and unsuccussed water controls (c0 + c1) for every individual experiment. Lines connecting data points are not interpolations.

Other substances of the screening (Crotalus horridus, Hepar sulfuris) were chosen with regard to the stressor arsenic and not specifically for one of the two organisms. The largest effects in the duckweed screening were obtained using homeopathically potentized Arsenicum album. The application of the latter substance does not seem to be specific to the organisms treated. Thus, comparability of the two systems regarding the choice of investigated substances was given in principle. Naturally, it is possible that some other potentized substances or potency levels might have induced larger effects in both the duckweed and yeast bioassay.

A potential advantage of the duckweed system is given due to the possibility of preselecting plants with a similar extent of impairment. While this selection is feasible for seeds up to a certain degree[30], it is impracticable for yeast. This might lead to an inhomogeneously impaired cell population with too few yeast organisms being damaged in a responsive range (i.e., not too strongly and not too weakly damaged).

It cannot be excluded that some special handling (e.g., mode of pipetting) in the yeast system or some specific feature of the experimental design prevented the occurrence of effects of the homeopathic preparations tested. However, our current hypothesis is that unicellular systems in general might yield less stable effects because of their huge ability for fast adaptation to multiple environmental impacts. Therefore, the influence of a single factor, like the effect of the homeopathic preparations, might be considerably reduced. In addition, higher organisms (e.g., plants, animals, humans) are of higher complexity than unicellular organisms, and exhibit more complex self-regulation and restoration processes. Higher organisms might be specifically susceptible towards the genuine nature of homeopathic preparations, e.g., in the case of an informational mode of action.

A difference in design between the two test systems exists regarding the mode of impairment. Because of the short generation time, yeast had to be stressed over the entire observation period. This led to a continuously decreasing stress due to the decreasing concentration of arsenic(V) in the nutrient solution caused by arsenic uptake of the growing and dividing yeast cells. In contrast, duckweed was stressed only before the homeopathic treatment. It might be favorable if stressor and homeopathic remedies would not act simultaneously. However, a counter-effect might occur at the end of the observation period in the duckweed system, when the plants are not impaired anymore. In this period, the homeopathic drug could act in the sense of a drug proving and could reverse the former effect of homeopathic remedies on impaired plants.

We know only one comparable investigation using impaired microorganisms in homeopathic basic research[31]. In this study, yeast (*Schizosaccharomyces pombe*) was stressed with $CuSO_4$ and treated with $CuSO_4$ in a potency range from 8x to 16x. No significant effects on growth were observed.

## CONCLUSION

Experiments with arsenic-impaired *Saccharomyces cerevisiae* showed no significant effects of the three tested homeopathic remedies (Arsenicum album, duckweed nosode, gibberellic acid) on growth of the test organism in this experimental setup. In contrast, the growth rate of arsenic-impaired *L. gibba* was enhanced after application of homeopathic potencies of Arsenicum album and duckweed nosode in repeated experiments. Thus, the experimental setup with *L. gibba* stressed by arsenic(V) and treated with Arsenicum album might be used as a valuable tool to investigate various topics of homeopathic basic research, e.g., external influences that probably affect the stability and quality of homeopathic preparations or investigations into the mode of action. The results obtained from the parallel yeast experiments can be interpreted in the sense that unicellular organisms may react to highly diluted homeopathic preparations in a less pronounced manner than multicellular organisms and seem to be a less useful tool for basic research into homeopathic potentization.

## ACKNOWLEDGMENTS

The authors thank Divya Pathak, Tanja Mendonça, and Roland Gosteli for laboratory assistance, as well as Silvia Ivemeyer for helpful comments. This investigation was funded by Weleda AG (Arlesheim, Switzerland). The sponsor had no influence whatsoever upon design, conduct, and evaluation of the investigation; the decision to publish; or the content of the manuscript. Additional financial support of the Karl und Veronica Carstens-Stiftung (Essen, Germany) and the Dr. Hauschka Stiftung (Bad Boll/Eckwälden, Germany) is gratefully acknowledged.
